# Melandrii Herba Extract Attenuates H_2_O_2_-Induced Neurotoxicity in Human Neuroblastoma SH-SY5Y Cells and Scopolamine-Induced Memory Impairment in Mice

**DOI:** 10.3390/molecules22101646

**Published:** 2017-09-30

**Authors:** Kwang Min Lee, Ae Sin Lee, Inwook Choi

**Affiliations:** Korea Food Research Institute, Seongnam-si, Gyeonggi-do 13539, Korea; kmlee@kfri.re.kr (K.M.L.); aslee@kfri.re.kr (A.S.L.)

**Keywords:** Melandrii Herba extract, neurotoxicity, oxidative stress, scopolamine, cell survival

## Abstract

Oxidative stress plays a significant role in the etiology of a variety of neurodegenerative diseases. In this study, we found that Melandrii Herba extract (ME) attenuated oxidative-induced damage in cells. Mechanistically, ME exhibited protection from H_2_O_2_-induced neurotoxicity via caspase-3 inactivation, Bcl-2 downregulation, Bax upregulation, and MAPK activation (ERK 1/2, JNK 1/2, and p38 MAPK) in vitro. Moreover, our in vivo data showed that ME was able to attenuate scopolamine-induced cognitive impairment. These results provide in vitro and in vivo evidence that ME exhibits neuroprotective properties against oxidative stress, which suggests that ME is worthy of further investigation as a complementary, or even as an alternative, product for preventing and treating neurodegenerative disorders.

## 1. Introduction

Oxidative stress has been implicated as a causative factor in neuronal damage, with such damage being one of the major causes of neurodegenerative disorders [1,2,3,4]. Reactive oxygen species (ROS) are generally highly reactive molecules, including oxygen radicals such as superoxide and hydroperoxyl radicals, and non-radical oxygen derivatives such as H_2_O_2_. These ROS induce oxidative stress, which can cause malfunctioning of DNA, proteins, mitochondria, and lipid membranes, and disrupt cellular function and integrity [5,6]. Among the various ROS, H_2_O_2_, which is one of the major agents generated by oxidative stress, is produced at nearly every stage of the oxidative cycle, and diffuses easily in and out of cells and tissues [7]. Neural cells exposed to H_2_O_2_ may undergo an apoptotic-like delayed death and necrosis. Numerous studies have implicated that oxidative stress plays a key role in the pathogenesis of neurodegenerative disorders [1,2,3,4,8,9,10,11] and have provided evidence that antioxidants can attenuate oxidative stress-induced neuronal cell damage [12]. Moreover, it has been demonstrated that neuronal cells are protected against oxidative stress-induced cell death by antioxidants such as polyphenols and flavonoids [13,14]. Therefore, antioxidants from natural products are thought to be substances capable of protecting normal neuronal cells from the oxidative stress-induced death or damage that leads to aging-related cognitive decline and neurodegenerative diseases [9,15,16,17,18].

Melandrii Herba is the unburied portion of fruiting *Melandryum firmum* Rohrbach (Caryophyllaceae). *M. firmum* is widely distributed in Asia and is used as a traditional Asian medicinal herb for treatment of gonorrhea, breast cancer, and anuria [19]. The main compounds in Melandrii Herba include sapogenins, saponins, triterpenoids, and flavonoids, which all exhibit bioactivity [20,21,22,23]. However, there has still been no clear evidence of the effect of Melandrii Herba on the fundamental cellular pathway that can explain its beneficial effects in a neuronal context. The main purpose of the current study was therefore to examine the neuroprotective properties of Melandrii Herba in an H_2_O_2_-induced cell death model of SH-SY5Y human neuroblastoma cells. In addition, in vivo benefits of Melandrii Herba were investigated in a scopolamine-induced memory impairment model.

## 2. Results

### 2.1. Melandrii Herba Extract Alleviated H_2_O_2_-Induced Damage in SH-SY5Y Cells

**Figure 1:**
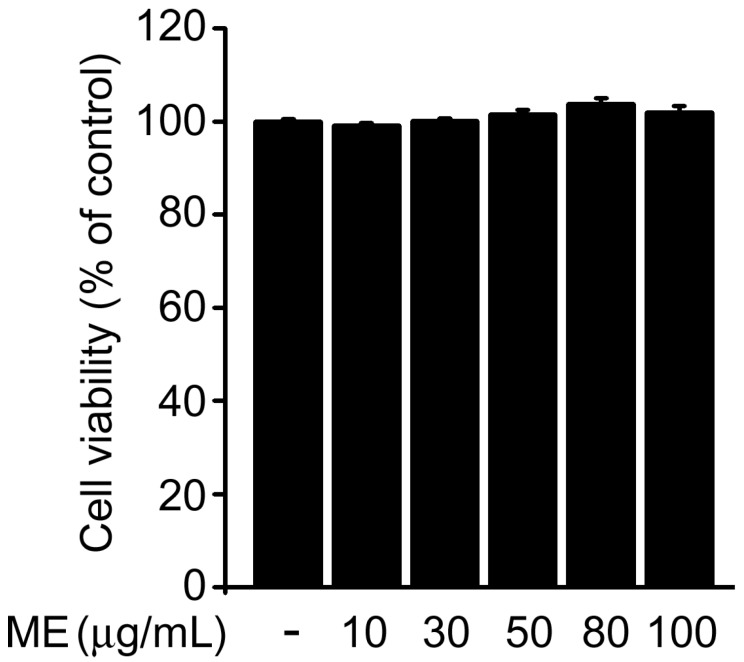
Cytotoxicity test of Melandrii Herba extract. Cells were exposed to the indicated concentrations of Melandrii Herba extract for 24 h. Cell viability was tested by MTT assay.

**Figure 2:**
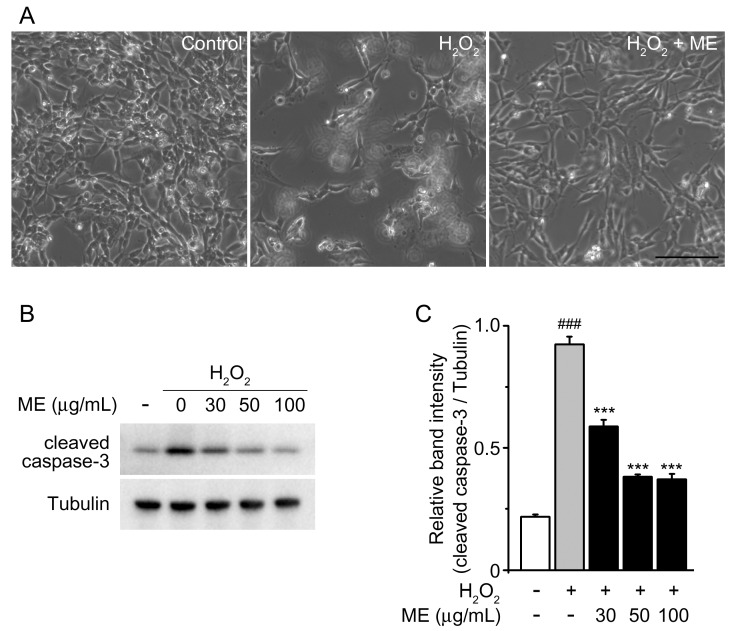
Effects of Melandrii Herba extract against H_2_O_2_-induced damage in cells. (**A**) Morphology of cells treated with H_2_O_2_ in the absence or presence of Melandrii Herba extract (100 ug/mL) for 24 h. Representative morphology was determined by phase-contrast microscopy. Normal morphology of SH-SY5Ycells was present in the control. Scale bar = 100 µm; (**B**) Western blots of endogenous cleaved caspase-3 in SH-SY5Y cells treated with the indicated dose of Melandrii Herba extract in the presence of H_2_O_2_ for 24 h. Tubulin was used as the loading control; (**C**) The ratio of cleaved caspase-3 to tubulin for the blot shown in (**B**). *** p < *0.005*, significantly different from the H_2_O_2_-treated control group. *^###^ p* < *0.005*, significantly different from the unstimulated control group.

We first used MTT assays to examine the possible cytotoxicity of Melandrii Herba extract (ME) in SH-SY5Y cells. ME treatment at concentrations of 10 to 100 µg/mL had no significant effect on cell viability compared with untreated controls (Figure 1). Next, we investigated the neuroprotective properties of ME in an H_2_O_2_-induced cell death model of SH-SY5Y cells. Morphological changes were observed in H_2_O_2_-treated cells as previously reported [24]; however, the H_2_O_2_-induced morphological changes were prevented by treatment with ME (Figure 2A).

Caspase-3 is a key mediator of cell death [25,26], and has been reported to be activated by H_2_O_2_ as a final effector of apoptotic cell death in vitro [27]. To confirm whether ME confers a neuroprotective effect against H_2_O_2_-induced cell death, we tested the level of endogenous cleaved caspase-3 protein in H_2_O_2_ and/or ME-treated cells. H_2_O_2_ treatment resulted in an increased level of endogenous cleaved caspase-3 in comparison with the untreated control group. However, incubation with ME reduced the H_2_O_2_-induced expression of endogenous cleaved caspase-3 in a dose-dependent manner. These results indicate that ME exhibited protection from H_2_O_2_-mediated cytotoxicity via the inhibition of caspase-3 activation.

### 2.2. Melandrii Herba Extract Inhibited H_2_O_2_-Induced Bax Upregulation and Bcl-2 Downregulation in SH-SY5Y Cells

**Figure 3:**
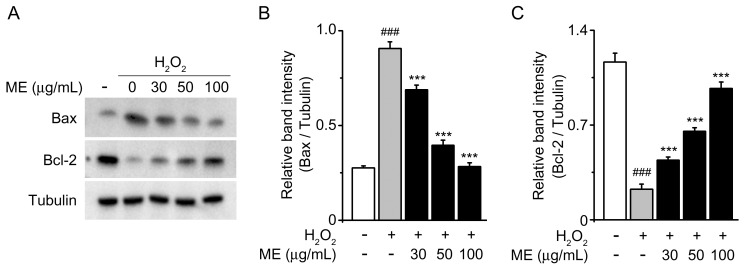
Effects of Melandrii Herba extract on the expression of Bax and Bcl-2 in H_2_O_2_ exposed cells. (**A**) Western blots of endogenous Bax and Bcl-2 in SH-SY5Y cells treated with the indicated dose of Melandrii Herba extract in the presence of H_2_O_2_ for 24 h. Tubulin was used as the loading control; (**B**) The ratio of Bax to tubulin; (**C**) The ratio of Bcl-2 to tubulin for the blot shown in (**A**). *** p < 0.005, significantly different from the H_2_O_2_-treated control group. *^###^ p < 0.005*, significantly different from the untreated control group.

To further explore the mechanism of the neuroprotective effects of ME, we investigated the protein expression levels of the pro-apoptotic Bax gene and the anti-apoptotic Bcl-2 gene, as they have crucial roles in modulating cell survival and cell death [28]. Decreased Bcl-2 expression and increased Bax expression were observed with the H_2_O_2_ treatment in comparison with the untreated control (Figure 3A, 1st & 2nd lanes). However, in those cells that were cultured with H_2_O_2_ and ME, the tendencies were reversed in a dose-dependent manner (Figure 3). These findings indicate that ME has the potential to inhibit H_2_O_2_-induced apoptosis.

### 2.3. Melandrii Herba Extract Prohibited H_2_O_2_-Stimulated MAPK Pathways

**Figure 4:**
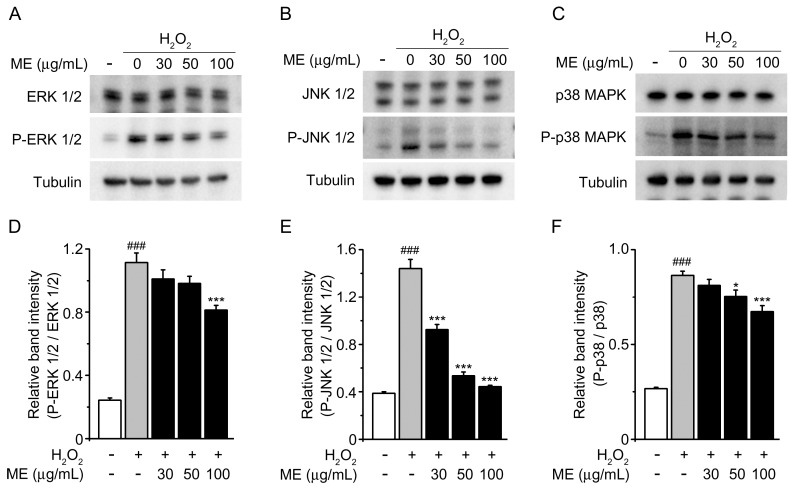
Effects of Melandrii Herba extract on ERK 1/2, JNK 1/2, and p38 expression in H_2_O_2_ exposed SH-SY5Y cells. (**A**) Western blots of endogenous ERK 1/2 and phosphor-ERK1/2 (P-ERK 1/2); (**B**) Western blots of endogenous JNK 1/2 and phosphor-JNK 1/2 (P-JNK 1/2); (**C**) Western blots of endogenous p38 MAPK and phosphor-p38 MAPK (P- p38 MAPK) in SH-SY5Y cells treated with the indicated dose of Melandrii Herba extract in the presence of H_2_O_2_ for 24 h. Tubulin was used as the loading control; (**D**) The ratio of P-ERK 1/2 to ERK for the blot shown in (**A**); (**E**) The ratio of P-JNK 1/2 to JNK 1/2 for the blot shown in (**B**); (**F**) The ratio of P-p38 MAPK to p38 MAPK for the blot shown in (**C**). * p < 0.05, *** p < 0.005, significantly different from the H_2_O_2_-treated control group. *^###^ p* < 0.005, significantly different from the unstimulated control group.

The aforementioned results raised a question regarding what potential pathways involved in apoptotic cell death were affected by ME. Numerous reports have elucidated that H_2_O_2_-induced oxidative stress may trigger cell death by activation of the MAPK family [29,30]. We therefore used Western blot analysis to examine MAPK activation and achieve an understanding of the mechanism of action underlying ME protection (Figure 4). After treating the cells with H_2_O_2_ alone, the phosphorylation of ERK 1/2, JNK 1/2, and p38 MAPK was significantly elevated compared with the untreated control. However, pretreatment with ME reduced the phosphorylation of ERK 1/2, JNK 1/2, and p38 MAPK. These results demonstrate that EM is able to protect cells against H_2_O_2_-induced oxidative stress through inhibition of the activation of MAPK pathway members.

### 2.4. Melandrii Herba Extract Attenuated Scopolamine-Induced Cognitive Impairment

**Figure 5:**
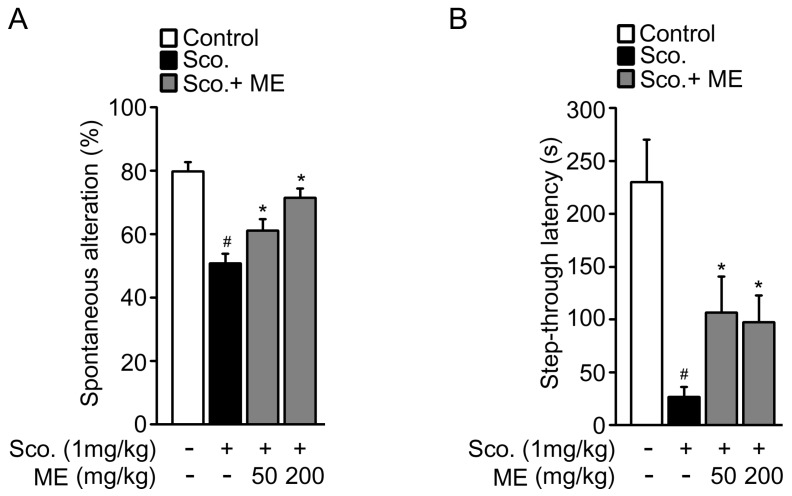
Effects of Melandrii Herba extract on scopolamine-induced cognitive impairment in mice. (**A**) Results from the Y-maze test (n = 7–8 per group); (**B**) Results from the passive avoidance test (n = 7–8 per group). * p < 0.05, significantly different from the scopolamine-treated control group. *^#^ p* < 0.05, significantly different from the untreated control group.

The pathogenesis of neurodegenerative diseases, including Alzheimer’s disease, Parkinson’s disease, and acute ischemic stroke, involves oxidative stress-induced neuronal cell death [1,2,3,4,8,9,10,11]. On the basis of these previous studies, we examined the in vivo benefits of ME in a scopolamine-induced memory impairment model. We first examined spatial working memory in a model system using a Y-maze test (Figure 5A). In this test, scopolamine-injected mice showed decreased spontaneous alternation compared with untreated control mice. The reduction in spontaneous alternation due to scopolamine was significantly reversed in a dose-dependent manner by supplementation with ME. We further performed a passive avoidance memory test to investigate the effects of ME on cognitive function (Figure 5B). The step-through latency of the scopolamine-injected mice was dramatically shorter than that of the saline-injected normal group. In the passive avoidance test, a lower latency time indicates the impairment of memory retention. Mice treated with both 50 mg/kg and 200 mg/kg of ME showed improvement in the step-through latency time in comparison with scopolamine-injected mice. Together, these results suggest that ME treatment diminished the scopolamine-induced impairment of learning and memory.

## 3. Discussion

Oxidative stress has been suggested as one of the major risk factors exacerbating neuronal loss in many neurodegenerative disorders, including Alzheimer’s disease, Parkinson’s disease, and Huntington’s disease. In this context, the search for potential neuroprotective agents from natural products that attenuate oxidative stress-induced neurotoxicity could be helpful in the prevention and treatment of neurodegenerative disorders [31].

In this study, we investigated the neuroprotective properties of Melandrii Herba (ME) using an H_2_O_2_-induced cell death model in SH-SY5Y cells. This cell line is commonly used as a model system for investigating neuronal cell death induced by oxidative stress, and for assessing the neuroprotective effects of natural products [32,33,34,35,36]. Numerous studies have used H_2_O_2_ (one of the major agents generated by oxidative stress) to induce neuronal damage, and a close association between H_2_O_2_ and neurodegenerative disorder has also been shown [10,11]. The main purpose of the current study was to investigate the effects of ME on oxidative stress-induced neuronal cell death, not to examine any specific neuronal diseases. Accordingly, we used H_2_O_2_ as an oxidative stress inducer, rather than any other specific stressor.

From our data, it was evident that neuronal cell death due to oxidative stress was significantly suppressed by ME treatment (Figure 2A). This result was further confirmed by immunoblotting for caspase-3, which is activated by H_2_O_2_ as an effector of apoptosis [27] (Figure 2B,C). Moreover, we observed that treatment with ME by itself demonstrated no cytotoxicity effect on SH-SY5Y cell viability (Figure 1). This observation is consistent with previous reports stating that oral doses of ME of up to 2000 mg/kg had no toxicity in vivo [37].

The Bcl-2 family has been well characterized as being involved in the apoptotic process [38]. When the proapoptotic homolog Bax is overexpressed in cells, apoptotic death in response to death signals is accelerated. Conversely, when Bcl-2 is overexpressed, it heterodimerizes with Bax, and cell death is repressed [28]. Our data revealed that increased Bax expression and decreased Bcl-2 expression by H_2_O_2_ were reversed by ME treatment. These results suggest that the neuroprotective effects of ME are involved in the balancing of Bax and Bcl-2 dependent apoptotic pathways.

We also considered the possible involvement of three subfamilies of MAPK in the neuroprotective effects of ME treatment: extracellular-signal regulated kinase 1/2 (ERK1/2), c-Jun NH2-terminal kinase (JNK), and p38 MAP kinase. These have been shown to be activated in response to the generation of ROS [29]. Moreover, H_2_O_2_ can rapidly activate ERK, JNK, and p38, which are all involved in the cell death induced by ROS. We observed that H_2_O_2_ activates MAPKs, which is in agreement with previous reports [36,39,40,41]; however, ME could effectively inhibit H_2_O_2_-induced phosphorylation of the three MAPKs, especially JNK (Figure 4). One possible interpretation is that JNK inactivation may be susceptible to ME treatment, allowing a degree of neuroprotection. Taken together, we conclude that ME has the ability to protect SH-SY5T cells against H_2_O_2_-induced cell loss.

Following on from our results demonstrating the neuroprotective effects of ME against oxidative stress in vitro, we performed mouse studies to understand the in vivo benefits of ME in a neuronal context. We chose a scopolamine-induced memory impairment mouse model that is commonly used as a screening system to assess the memory-enhancing properties of substances [42,43,44,45]. Our in vivo data suggested that ME could attenuate scopolamine-induced cognitive impairment (Figure 5). Recent studies have shown that cognitive impairment in the scopolamine-induced animal model is associated with increased oxidative stress within the brain [46,47,48]. The in vivo functional roles of ME require further elucidation; however, in consideration of these recent reports, our in vivo findings could be interpreted as indicating that at least a part of the in vivo benefits come from its neuroprotective effects against oxidative stress.

## 4. Materials and Methods

### 4.1. Cell Culture and Treatment

Human neuroblastoma SH-SY5Y cell lines were cultured in Dulbecco’s modified Eagle’s medium (DMEM, GIBCO) with 10% (*v*/*v*) fetal bovine serum (FBS, Hyclone) and 1% penicillin/streptomycin at 37 °C in a humidified 5% CO_2_ atmosphere. Cells were pretreated with various concentrations of Melandrii Herba extract for 1 h and then exposed to H_2_O_2_ (50 µM) for 24 h.

### 4.2. Preparation of Melandrii Herba Extract (ME)

The dried Melandrii Herba was kindly provided by Gyungdong Herbal Market (Seoul, Korea) and was extracted by fermented ethanol with the assistance of sonication at room temperature for 1 h. The extracted solution was then filtered using filter paper (Whatman, Piscataway, NJ, USA) and concentrated with a rotary vacuum evaporator (Buchi, Tokyo, Japan). The fermented ethanol was removed and extracts were lyophilized. The extracts were freeze-dried and kept at −70 °C until use.

### 4.3. Western Blotting

Proteins were separated by sodium dodecyl sulfate polyacrylamide gel electrophoresis (SDS-PAGE) and blotted onto polyvinylidene fluoride (PVDF) membranes. After blocking with 3% bovine serum albumin (BSA) in Tris-buffered saline containing 0.2% Tween-20 (TBS-T), the blots were incubated with various primary antibodies, including anti-cleaved caspase-3, anti-Bax, anti-Bcl-2, anti-p44/42 mitogen-activated protein kinase (MAPK; Erk1/2), anti-phospho-p44/42 MAPK (Erk1/2), anti-p38 MAPK, anti-phospho-p38 MAPK, anti-JNK, anti-phospho-JNK, and anti-tubulin. The blots were then incubated with secondary antibody [anti-rabbit horseradish peroxidase (HRP)-conjugate or anti-mouse HRP-conjugate (Santa Cruz Biotechnology, Dallas, TX, USA)] and the protein bands were visualized using an enhanced chemiluminescence detection system (ECL; Amersham Pharmacia, Piscataway, NJ, USA).

### 4.4. Experimental Animals

Male ICR mice (6-week-old) were used in this study. Animals were maintained under pathogen-free conditions. All experiments were approved by the Institutional Animal Care and Use Committee (IACUC) of the Korea Food Research Institute. For in vivo treatments, the mice were orally treated with either normal saline or ME once daily for 4 weeks. Memory impairment was induced by treatment with scopolamine (1 mg/kg, i.p.), and either a spatial memory test or a passive avoidance test was performed 30 min after the treatment.

### 4.5. Spatial Memory Test

A Y-shaped maze with three arms was made from black Plexiglas (40 × 4 × 13 cm). Spontaneous alternation was tested as described previously [49,50,51,52]. For the tests, animals were placed in the end of one arm and allowed to move freely through the maze for 7 min in dim light. An entry was recorded as the placing of all four paws into an arm. The percentage of spontaneous alternations was calculated as the ratio of the number of successful alternations to the number of total alternations minus 2.

### 4.6. Passive Avoidance Test

The passive avoidance test was performed as previously described [51,52,53]. In brief, the passive avoidance apparatus consisted of a light and dark chamber separated by a guillotine door. The floor of the dark chamber was made of stainless-steel grids. During habituation, mice were allowed to freely explore the box for 5 min with the door open and were then returned to their home cage. For conditioning, which was performed after 24 h, the mice were placed into the light chamber and the sliding door was closed when both hindlimbs of a mouse were within the dark chamber. An electric foot shock (0.3 mA, 3 s) was then delivered through the floor grids. Ten seconds later, the mice were returned to their home cage. Tests were carried out 24 h after the conditioning, and the latency time for mice to enter the dark chamber was measured using a 300 s cut-off.

### 4.7. Statistical Analysis

All displayed values represent means ±SEM. Significant differences between groups were determined using two-tailed unpaired Student’s *t*-tests, and multiple comparisons were performed using one-way ANOVA or two-way repeated-measures ANOVA. Differences with *p* < 0.05 were considered statistically significant and are indicated in the figure legends.

## 5. Conclusions

In summary, this study provides the first in vitro and in vivo evidence that Melandrii Herba extract (ME) exhibits neuroprotective properties against oxidative stress. Our present findings suggest that ME is worthy of further investigation as a complementary, or even an alternative, product for preventing and treating neurodegenerative diseases.
